# Arginine Methylation of the PGC-1α C-Terminus Is Temperature-Dependent

**DOI:** 10.1021/acs.biochem.2c00363

**Published:** 2022-12-19

**Authors:** Meryl Mendoza, Mariel Mendoza, Tiffany Lubrino, Sidney Briski, Immaculeta Osuji, Janielle Cuala, Brendan Ly, Ivan Ocegueda, Harvey Peralta, Benjamin A. Garcia, Cecilia I. Zurita-Lopez

**Affiliations:** † Department of Chemistry and Biochemistry, California State University, Los Angeles, 5151 State University Drive, Los Angeles, California 90033, United States; ‡ Department of Biochemistry and Biophysics, University of Pennsylvania, Philadelphia, Pennsylvania 19104, United States; § Department of Biochemistry and Molecular Biophysics, Washington University School of Medicine, St. Louis, Missouri 63110, United States; ∥ Schmid College of Science and Technology, Keck Center for Science and Engineering, Chapman University, 450 N. Center Street, Orange, California 92866, United States

## Abstract

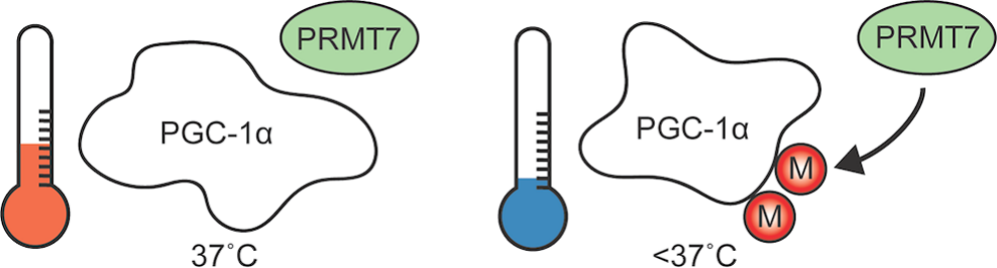

We set out to determine whether the C-terminus (amino acids 481–798) of peroxisome proliferator-activated receptor gamma coactivator-1 alpha (PGC-1α, UniProt Q9UBK2), a regulatory metabolic protein involved in mitochondrial biogenesis, and respiration, is an arginine methyltransferase substrate. Arginine methylation by protein arginine methyltransferases (PRMTs) alters protein function and thus contributes to various cellular processes. In addition to confirming methylation of the C-terminus by PRMT1 as described in the literature, we have identified methylation by another member of the PRMT family, PRMT7. We performed *in vitro* methylation reactions using recombinant mammalian PRMT7 and PRMT1 at 37, 30, 21, 18, and 4 °C. Various fragments of PGC-1α corresponding to the C-terminus were used as substrates, and the methylation reactions were analyzed by fluorography and mass spectrometry to determine the extent of methylation throughout the substrates, the location of the methylated PGC-1α arginine residues, and finally, whether temperature affects the deposition of methyl groups. We also employed two prediction programs, PRmePRed and MePred-RF, to search for putative methyltransferase sites. Methylation reactions show that arginine residues R548 and R753 in PGC-1α are methylated at or below 30 °C by PRMT7, while methylation by PRMT1 was detected at these same residues at 30 °C. Computational approaches yielded additional putative methylarginine sites, indicating that since PGC-1α is an intrinsically disordered protein, additional methylated arginine residues have yet to be experimentally verified. We conclude that temperature affects the extent of arginine methylation, with more methylation by PRMT7 occurring below physiological temperature, uncovering an additional control point for PGC-1α.

## Introduction

Peroxisome proliferator-activated receptor gamma (PPARγ) coactivator-1 alpha (PGC-1α) is a transcriptional coactivator capable of forming complexes with transcription factors such as NRF-1, NRF-2, PPARα, PPARδ, PPARγ, ERRα, and TR.^1^ It has regulatory functions in lipid metabolism, mitochondrial biogenesis, remodeling of muscle tissue, and more recently inflammatory response pathways.^2−4^ Initially, PGC-1α was identified as a thermoregulator, whose expression was induced upon exposure to cold temperatures (4–24 °C).^5^ However, PGC-1α is now implicated in diseases such as type 2 diabetes and obesity,^6,7^ cancer,^8,9^ and neurodegenerative diseases such as Parkinson’s^10^ and Huntington’s^11^ disease. Given its various functions and significance, how this protein is regulated is the subject of intense investigation.

According to PhosphoSitePlus, PGC-1α is not only heavily phosphorylated but also post-translationally modified with ubiquitin, acetyl, and methyl groups.^12^ With respect to methylation, human PGC-1α becomes methylated at arginine residues 665, 667, and 669 (within RS and E regions of the C-terminus) at 30 °C by protein arginine methyltransferase 1 (PRMT1).^13^ PRMT1 is the most active member of a family of nine PRMT enzymes that methylate arginine residues.^14,15^ Arginine methylation at 665, 667, and 669 by PRMT1 was found to decrease the expression of the ERRα promoter, which is important for mitochondrial biogenesis.^13^ In addition, this same study found additional methylation within the C-terminus; however, the exact arginine residues and the methyltransferase(s) responsible for this methylation were not identified.^13^ Since PRMT1 is one of nine members of this family of cellular regulators,^16^ we hypothesized that there are other PRMTs that contribute to the regulation of PGC-1α *via* arginine methylation, in particular PRMT7. Of the nine mammalian protein arginine methyltransferases, PRMT1 (and not CARM1/PRMT4) methylates PGC-1α.^13^ PRMT1 and CARM1/PRMT4 are the most active methyltransferases that produce ADMA. In addition, to our knowledge, there is no evidence of SDMA in PGC-1α, and thus we focused on PRMT7.

PRMT7 is a unique member of the methyltransferase family. Not only is it larger than the rest of the family members, but it is also the only known type III enzyme, capable of solely producing ω-monomethylated arginine (ω-MMA) residues.^17−19^ Moreover, it preferentially methylates arginine residues found in RXR motifs (where R represents arginine, and X represents any amino acid). PRMT7 shows the greatest activity when substrates contain RXRXR motifs as seen in histone H2B.^18,20^ PGC-1α contains various RXR motifs. Finally, PRMT7 is also sensitive to temperature.^17,20^

We set out to determine whether the canonical isoform of PGC-1α (UniProt Q9UBK2) is a substrate for PRMT7. Since previous work by Teyssier et al. shows that PGC-1α is methylated in the C-terminus, we focused on this portion of the protein (amino acids 481–798). *In vitro* methylation reactions were performed where the C-terminus of PGC-1α was incubated with recombinant mammalian PRMT7 enzyme. Fluorography assays and mass spectrometry were used to assess arginine methylation. We also employed computational methylation prediction programs to search for additional putative methylated arginine residues. Our *in vitro* findings demonstrate that both PRMT1 and PRMT7 methylate arginine residues at temperatures at or below 30 °C. In addition to demonstrating that PGC-1α is a substrate for PRMT7, we also identified novel methylated arginine residues by PRMT1. Our *in silico* studies indicate that PGC-1α is capable of receiving additional methyl groups at arginine residues, perhaps by additional members of the methyltransferase family, but whose exact conditions remain to be discovered. Our results provide novel insights into the regulation of this protein.

## Materials and Methods

### Protein Expression and Purification

Wild-type constructs of PRMT1 and a PGC-1α plasmid known as G1 were transformed from DH5α to BL21 *Escherichia coli* cells. For a list of the PGC-1α constructs used, including their amino acid sequences, see Table S1. All constructs were streaked on ampicillin (Amp) plates (100 mg/mL) and were bacterially expressed as described previously with the exception of PRMT7.^21^ To optimize protein expression for active PRMT7, a starter culture was selected from a single colony and used to inoculate 25 mL of YT medium (Amp 100 mg/mL) and incubated in a 37 °C shaker for 10–12 h.^22^ This culture (20 mL) was transferred to 450 mL of Terrific broth, 4 mL of 50% glycerol, and 50 mL of 10 × buffering salt (2.31 g KH_2_PO_4_, 16.4 KH_2_PO_4_·3H_2_O for 100 mL) in a 1 L Erlenmeyer flask. The samples were incubated for approximately 4–5 h at a 37 °C until the optimum density (OD) reached an absorbance of 0.6–0.8 and induced with a final concentration of 1.0 mM isopropyl β-d-1-thiogalactopyranoside (IPTG) overnight at 16 °C.

The cells were harvested in centrifuge bottles and spun at 6000*g* for 8 min at 4 °C. The pellet was dissolved and collected with 25 mL of 1 × phosphate-buffered saline (PBS) buffer and spun down again at 5000*g* for 5 min at 4 °C. Once expressed, all proteins were purified. Briefly, the pellet was thawed and dissolved in 8 mL of 1 × PBS in the presence of 80 μL of 1 M phenylmethylsulfonyl fluoride (PMSF). The GST-proteins were released *via* bacterial cell sonication with seven cycles of 20 s pulses with a 1-min break in between each pulse. Following sonication, the samples were centrifuged at 23,000*g* for 50 min at 4 °C. An additional 80 μL of 1 M PMSF was added to prevent protein degradation.

GST-PRMT7 was purified as per the manufacturer’s specifications using glutathione Superflow Agarose (PierceTM Glutathione Superflow Agarose Thermo Scientific Protocol). Briefly, the protein extraction was added to the prepared agarose and mixed on a rotator for 2 h at 4 °C. The solution was centrifuged for 2 min at 700*g* and was washed four times with 2 resin-bed volumes of equilibration buffer (125 mM Tris–HCl, 150 mM sodium chloride; pH 8.0). The GST-tagged proteins were eluted with 1 resin-bed volume of elution buffer (125 mM Tris–HCl, 150 mM sodium chloride, 10 mM reduced glutathione; pH 8.0) and mixed slowly for 10 min. The sample was spun for 2 min at 700*g* at 4 °C. Eluent fractions were stored at −80 °C. With respect to GST-PRMT7 expression and purification, the samples were immediately used for methylation reactions to minimize protein degradation and subsequent loss of enzymatic activity. Protein concentration was determined as previously described *via* TCA Lowry assay using bovine serum albumin (BSA) 1 mg/mL as a standard.^23^

### In Vitro Methylation Reactions

An enzyme, either PRMT1 or PRMT7, was incubated with either bacterially expressed construct of PGC-1α: G1 (amino acids 566–640); the C-terminus of PGC-1α (AbCam, amino acids 481–798, His tag C-terminus); or a fragment of the C-terminus PGC-1α (Creative Biomart, amino acids 573–767, His tagged C-terminus) in the presence of 0.5 μM *S*-adenosyl-l-[methyl-^3^H]methionine and of 50 mM 4-(2-hydroxyethyl)-1-piperazineethanesulfonic acid (HEPES), 10 mM NaCl, and 1 mM dithiothreitol (DTT) for 1 h at various temperatures ranging from 4 to 37 °C in a final volume of 30 μL. For fluorography, the reactions were immediately quenched with 4 × loading dye (100 μL of 2-mercaptoethanol per 950 μL of Laemmli sample buffer; BioRad), resolved *via* a 12% sodium dodecyl sulfate poly(acrylamide) gel electrophoresis (SDS-PAGE) and stained with Coomassie Blue. Next, the gel was incubated with enhance (PerkinElmer) for an hour and placed in a 10% glycerol solution for an additional hour on a nutator. The gel was then dried and exposed on film at −80 °C for various lengths of time. The film was developed with a developer and fixer solution (GBX Developer and Fixer, M&S Dental, New York).

Nonradioactive *in vitro* methylation reactions were carried out in a similar way except that reactions were incubated in the presence of a final concentration of 3.2 mM *S*-adenosylmethionine (AdoMet) (New England Biolabs, Inc.). These samples were analyzed by mass spectrometry.

### In-Gel Digestion and Peptide Extraction for Mass Spectrometry Analyses of Methylated PGC-1α Products

Nonradioactive methylation reactions were resolved *via* 12% SDS-PAGE gel and stained with Coomassie blue as described above. After destaining, the gel bands of interest were sliced out and diced into 1 mm slices on a clean glass plate and placed in a microcentrifuge tube. The gel slices were rinsed with 100 mM ammonium bicarbonate (ABC), then destained completely in 50% acetonitrile/100 mM ABC for 1 h on a nutator and further dried on a speed vacuum concentrator (speed vac). Samples were reduced with 10 mM DTT in 100 mM ABC for 1 h at 56 °C, then were alkylated with 55 mM iodoacetamide in 100 mM ABC for 45 min in the dark. After alkylation, the gel slices were washed with 100 mM ABC, dehydrated with 100% acetonitrile, and dried by speed vacuum. The slices were then incubated with 12.5 ng/μL trypsin diluted in 100 mM ABC with enough volume to completely cover them, placed on ice for 20 min, then left on the bench overnight.

After overnight trypsin digestion, peptides were extracted from the gel slices *via* a series of hydration and dehydration steps using ABC and acetonitrile solutions. Briefly, gel slices were rinsed with 100 mM ABC for 45 min and the supernatant was collected. Next, gel slices were dehydrated with a 50:45:5 ratio of acetonitrile, water, and acetic acid for 15 min to inactivate trypsin and rehydrate with 100 mM ABC for 15 min. After a subsequent round of hydration, dehydration, and hydration, the gel slices were dehydrated with 100% acetonitrile until the gel slices became white. All of the supernatant collected from the extraction was pooled and dried in a speed vac. The samples were then desalted using a C18 column as previously described.^24^

Dried samples were resuspended in buffer A (0.1% (v/v) formic acid in water) and loaded into a Nano-LC system (EASY-nLC 1000, Thermo Fisher Scientific) coupled online with an Orbitrap Fusion Tribrid mass spectrometer (Thermo Scientific). Peptides were separated on a home-packed capillary column (200 mm length, 75 μm inner diameter) containing reverse-phase ReproSil-Pur C18-AQ resin (3 μm particle size, Dr. Maisch Gmbh) at a flow rate of 300 nL/min. A gradient of 20 min was set from 5 to 35% buffer B (0.1% formic acid in acetonitrile), then 35–98% buffer B in 12 min. Full scan mass range of *m*/*z* 250–1100 was analyzed in the Orbitrap at 120,000 resolution and 5.0 × 10^5^ AGC target value. MS/MS was performed in the Orbitrap at 30,000 resolution in the normal mode using data-dependent acquisition. The HCD collision energy, AGC target, and maximum injection time were set to 27, 5.0 × 10^4^, and 50 ms, respectively. Dynamic exclusion (20 s) was enabled. Every sample was injected once into Orbitrap Fusion.

### Mass Spectrometry Data Processing and Analysis

MS raw files of proteome analyzed by Proteome Discoverer 2.1 software against a modified Fasta file that only includes the proteins used for the *in vitro* reactions: PRMT1 (Uniprot ID Q99873), PRMT7 (Uniprot ID Q9NVM4), and the truncated PGC-1α. Additionally, the database that included common contaminants was used for the first search. Trypsin was specified as the digesting enzyme with two missed cleaves allowed. The search included fixed modifications of carbamidomethyl cysteine and variable modifications of methionine oxidation, N-terminal acetylation, methyl (KR), and dimethyl (KR).

In addition, an initial screen of methylation reactions was carried out and analyzed by mass spectrometry at the Mass Spectrometry Facility at UC Irvine. Briefly, nonradioactive *in vitro* methylation reactions with either PRMT7 or PRMT1 and PGC-1α fragments (C-term, Creative Biomart) were incubated with AdoMet for 1 h at temperatures 37, 30, 21, 18, and 16 °C. Samples were then quenched with 2 × loading dye, resolved on 12% SDS-PAGE, and then stained as described above. Methylated PGC-1α bands and controls with PGC-1α, PRMT1, and PRMT7 alone were submitted to UC Irvine Mass Spectrometry Facility for analyses. Raw files for all MS proteome work have been deposited to the Chorus repository (https://chorusproject.org/pages/index.html) under project number 1765.

### Methylation Predication Programs

To search for putative methylated arginine residues computationally, the full-length Fasta amino acid sequence for PGC-1α (UniProt Q9UBK2) was imputed into PRmePRed^25^ and MePred-RF^26^ arginine prediction programs. Settings for PRmePRed: window size 19 amino acids and a 0.5 support vector machine (SVM) threshold. Settings for MePred-RF: window size 11 amino acids and a 0.5 random forest (RF) threshold. The putative arginine residues with overlapping arginine residues in PGC-1α on a Venn diagram were created using Canva (canva.com).

## Results and Discussion

PGC-1α has various protein partners and is found in tissues with high energy demands that are rich in mitochondria such as muscle, liver, heart, and pancreas.^27−30^ To investigate this protein, we set out to determine whether PGC-1α is methylated at the C-terminus by PRMT7, which is also widely expressed in tissues.^31^ We focused on the C-terminal portion of this protein because to date, it is the only portion found to be methylated at arginine residues.^8,13^

**Figure 1:**
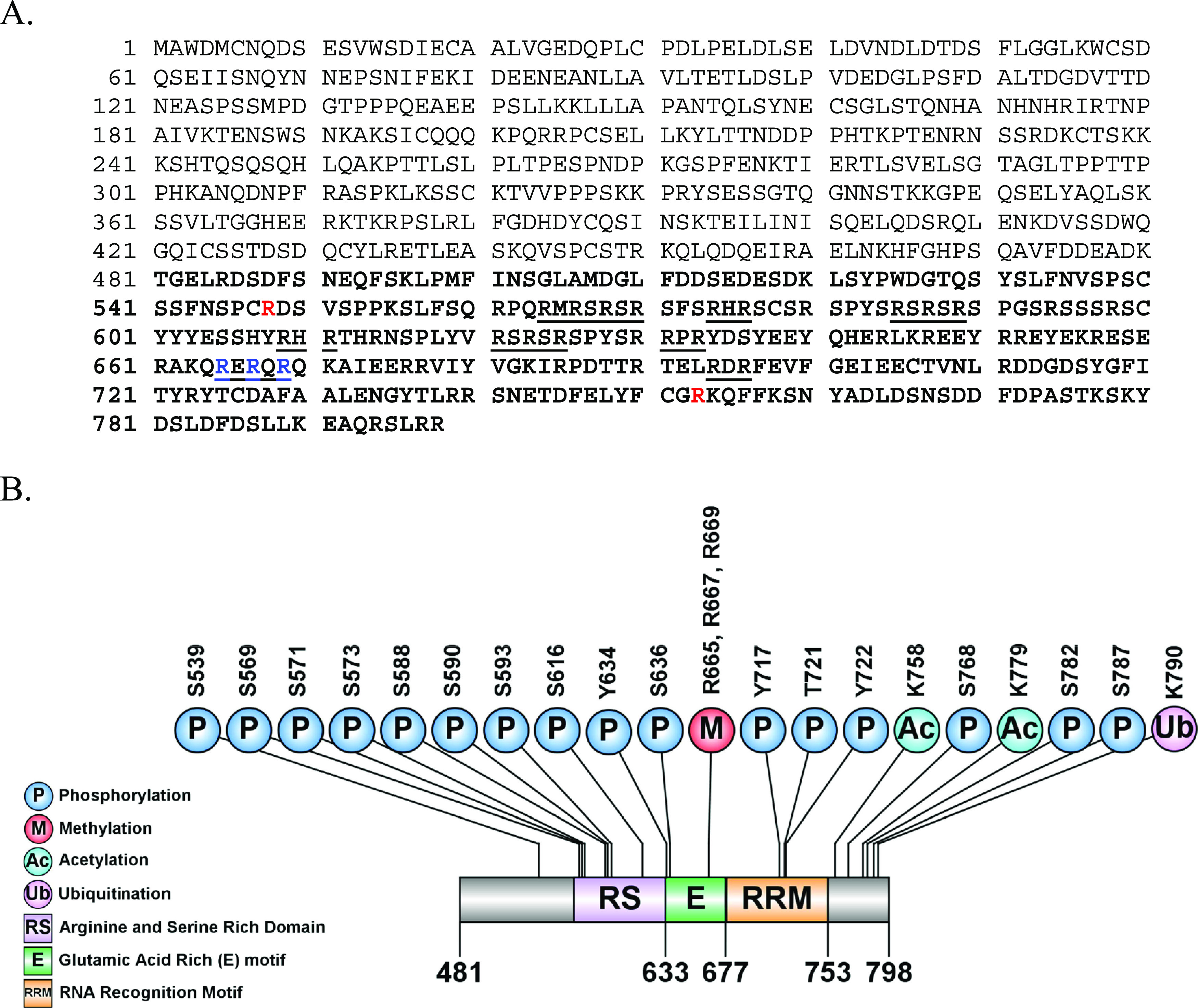
(A) Full-length human PGC-1α protein sequence (UniProt Q9UBK2; MW 91 kDa), C-terminus in boldface (amino acids 481–798, predicted MW 38 kDa), methylated arginine residues confirmed experimentally in this study: R548 and R753 (red), additional (previously confirmed) methylated arginine residues R665, R667, and R669 (blue).^13^ Four RXR, three RXRXR, and one RXRXRXR arginine-rich regions are underlined. (B) Map of PGC-1α with identified PTMs located in the C-terminus. PTMs were identified using the following databases: dbPTM^40^ and PhosphoSitePlus.^12^ Created using Illustrator for Biological Sequences (IBS).^41^

PGC-1α is asymmetrically dimethylated by PRMT1 at arginine residues 665, 667, and 669. This modification is an activating mark that promotes the expression of target genes important for mitochondrial biogenesis.^13^ Although PRMT1 methylates PGC-1α, knockdown does not completely abolish all arginine methylation in the C-terminus of PGC-1α purified from COS7 cells.^13^ We searched for evidence of PGC-1α being methylated or associating with any other PRMT family member. We found two studies where PRMT5 modulates the expression of PGC-1α in hepatocytes,^32,33^ and three studies where PRMT5 expression is increased during muscle plasticity.^34−36^ In addition, another study found that the expression of PRMT7 increases the expression of PGC-1α.^37^ Since to date there is no evidence of PGC-1α receiving an SDMA mark (catalyzed by PRMT5), we reasoned that in addition to PRMT1, PRMT7 may also methylate PGC-1α. Moreover, PGC-1α contains four RXR regions, three RXRXR regions, and one RXRXRXR arginine-rich region within the C-terminus (Figure 1A), which could be potential sites for PRMT7 methylation. The amino acid sequence of PGC-1α (UniProt Q9UBK2) is shown in Figure 1A, where the C-terminus is in boldface and the methylated arginine residues verified experimentally in this study are identified in red. PGC-1α contains an RS domain (rich in arginine and serine residues, aa 565–598 and 617–631), an acidic E region (rich in glutamic acid residues, aa 632–676), and an RNA binding domain (RBD) which is a common PRMT substrate also known as an RNA recognition motif (RRM) (aa 677–753).^38,39^Figure 1B emphasizes the C-terminus of PGC-1α, highlighting various post-translational modifications (PTMs) identified to date.^12,40,41^

**Figure 2:**
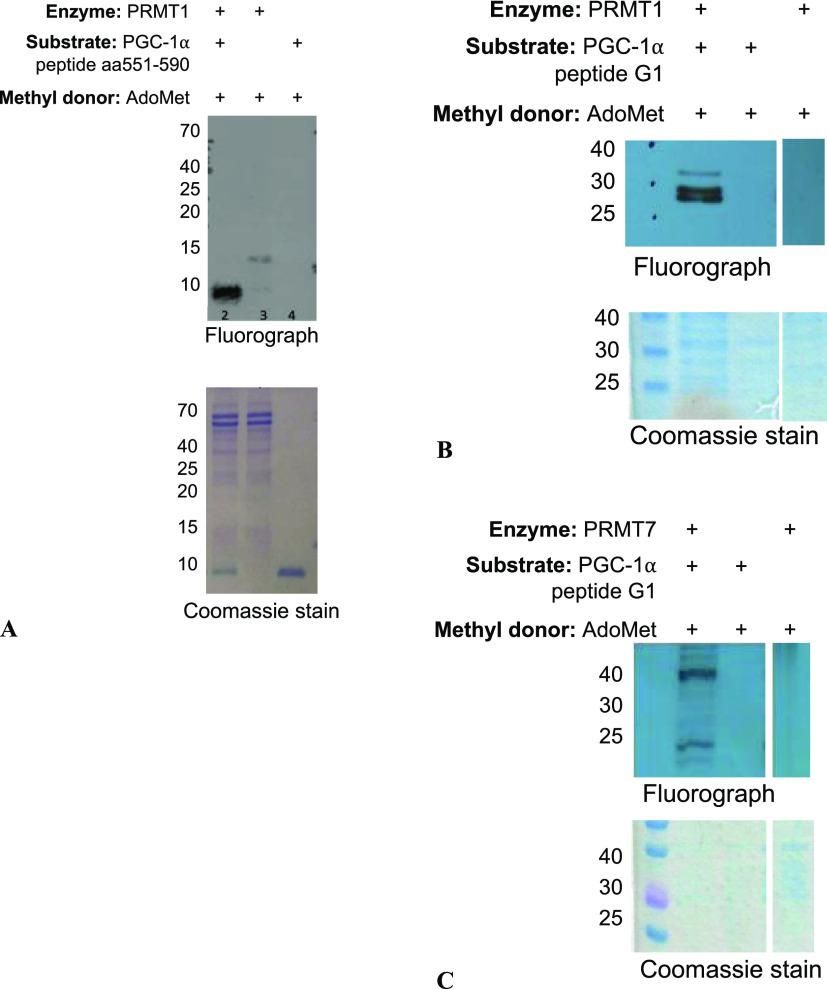
(A) Methylation of a peptide corresponding to PGC-1α sequence VSPPKSLFSQRPQRMRSRSRSFSRHRSCSRSPYSRSRSRS (aa 551–590; ∼4.7 kDa). Recombinant GST-PRMT1 (4 μg) was incubated with peptide (5 μg) in the presence of 0.5 μM *S*-adenosyl-l-[methyl-^3^H]methionine for 1 h at 37 °C with 9 μL of 10 × HEPES buffer in a final volume of 90 μL as described in the Materials and Methods section. The samples were then resolved on a 15% SDS-PAGE gel (lower panel). The radioactive methylation reactions were exposed on film as described in the Materials and Methods section for 3 weeks (upper panel). (B) Methylation of G1 corresponding to PGC-1α sequence (aa 532–640; ∼38 kDa). Recombinant GST-PRMT1 (7 μg) was incubated with G1 (7.6 μg) in the presence of 0.5 μM *S*-adenosyl-l-[methyl-^3^H]methionine for 1 h at 37 °C with 3 μL of 10 × HEPES buffer in a final volume of 30 μL as described in the Materials and Methods section. The samples were then resolved on a 15% SDS-PAGE gel (lower panel). The radioactive methylation reactions were exposed on film as described in the Materials and Methods section for 5 days (upper panel). (C) Recombinant GST-PRMT7 (5 μg) was incubated with GST-G1 (5 μg) in the presence of 0.5 μM *S*-adenosyl-l-[methyl-^3^H]methionine for 20 h at 22 °C with 4 μL of 10 × HEPES buffer in a final volume of 40 μL as described in the Materials and Methods section. The samples were then resolved on a 12% SDS-PAGE gel (lower panel). The radioactive methylation reactions were exposed on film as described in the Materials and Methods section for 1 month (upper panel). The GST-G1 construct is 108 amino acids long or approximately 11 kDa. The GST has a molecular weight of approximately 27 kDa, making G1 approximately 38 kDa.^13^

*In vitro* methylation reactions were carried out using portions of the C-terminus corresponding to PGC-1α as substrates. We included a small peptide (∼40 aa) as a substrate that corresponds to amino acids 551–590, a portion of PGC-1α that contains 11 arginine residues many of which are part of an RXR motif (for peptide sequence and a list of all substrates, see Table S1). To screen for methylation, purified recombinant GST-PRMT1 was used to methylate this small peptide as well as a bacterially expressed GST construct of PGC-1α known as G1. Figure 2 shows that PRMT1 can methylate the small peptide (Figure 2A) and G1 construct (Figure 2B) at 37 °C. PRMT7 is able to methylate the PGC-1α GST-G1 construct at 22 °C (Figure 2C).

**Figure 3:**
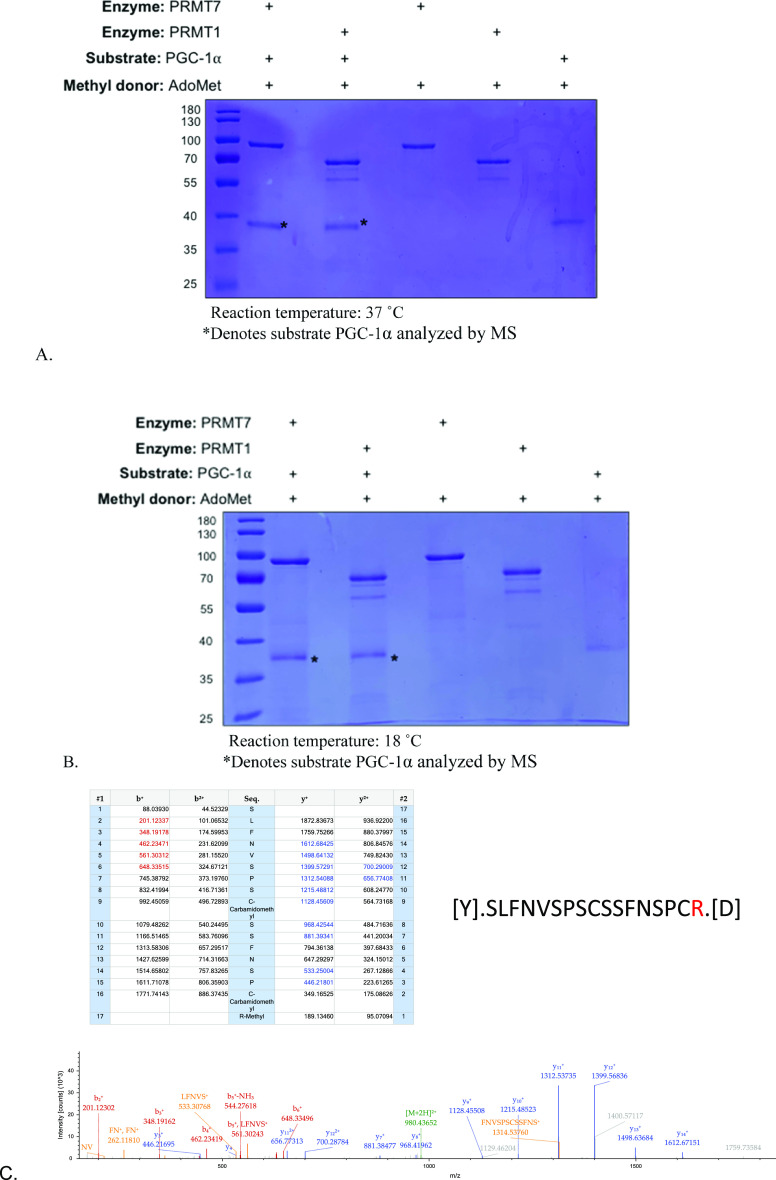
(A) Arginine methylation of PGC-1α (2 μg) (573–767) at (A) 37 °C and (B) 18 °C by 2 μg of either PRMT1 or PRMT7. Substrate was mixed with 32 mM AdoMet in 50 mM HEPES, 10 mM NaCl, and 1 mM DTT in a final volume of 150 μL master mix. Aliquots were then incubated at 37, 30, 21, 18, 16, and 4 °C for 1 h followed by the addition of enzyme. The samples were then quenched with 4 × loading dye and resolved on a 12% SDS-PAGE gel as described in the Materials and Methods section. (C) Detection of methylated arginine peptide fragment (sequence SLFNVSPSCSSFNSPCR) corresponding to PGC-1α by mass spectrometry. Isolation of this 2+ charge species is denoted by a green peak (980.43652 *m*/*z*, monoisotopic mass 1960.97; calculated mass 1831.8102 without methyl group or carbamidomethylation of cysteine residues (57.02145 Da) due to iodoacetamide). The table shows the fragmentation patterns of the b and y ions. Red and blue colors indicate fragments identified.

Since our fluorographs show that PRMT7 methylates the C-terminal region of PGC-1α, we decided to investigate whether temperature affects methylation in this region. PGC-1α is highly induced in brown fat and skeletal muscle in mice kept at 4 °C.^5^ It is a cold-inducible coactivator associated with adaptive thermogenesis, an important component of energy homeostasis.^2,42^ More recently, PRMT7 has also shown sensitivity to temperature. It is most active below room temperature with less than 10% activity at 37 °C *in vitro*.^17,20,43^ Moreover, unlike other members of the PRMT family, PRMT7 (and PGC-1α) is relatively tolerant to low temperatures and sensitive to high temperatures.^18,27^ We performed *in vitro* methylation reactions using commercially purchased constructs corresponding to the C-terminus of PGC-1α and incubated the reactions at the following six temperatures: 37, 30, 21, 18, 16, and 4 °C. Figure 3A,B shows representative SDS-PAGE gels where reactions were performed at 37 and 18 °C (for panels of methylation reactions performed at other temperatures, see the Supporting Information). To specifically localize arginine methylation, we excised the gel bands corresponding to the methylated substrate and performed LC-MS/MS analyses. Figure 3C shows a representative spectrum of a fragmented species corresponding to residues 532–548 of PGC-1α. The mass spectrum is one of three fragments denoting methylarginine R548 detected in this same region (for additional spectra, see the Supporting Information).

Methylated peptide fragments obtained after cutting out SDS-PAGE bands that correspond to PGC-1α were analyzed by LC-MS/MS. Over 20 samples corresponding to methylation reactions and including controls (gel background) were analyzed and summarized in Table 1. Specifically, monomethylation is detected at residues R548 and R753 by PRMT1 at 30 °C, at R548 at 21 °C and at residue R753 at 4 °C. Monomethylation is detected at residues R548 and R753 by PRMT7 at 30 °C, and at R548 at 18 °C. Methylation reaction controls, where both PRMT1 and PRMT7 are incubated without the substrate PGC-1α, are methylated regardless of temperature, indicating that temperature affects the PGC-1α substrate and does not hinder the enzyme’s ability to automethylate. This is consistent with studies that show the stability of PRMTs at nonphysiological temperatures.^44^ Methylation of PGC-1α by both PRMT1 and PRMT7 is greater at temperatures at or below 30 °C.

**Table 1:**
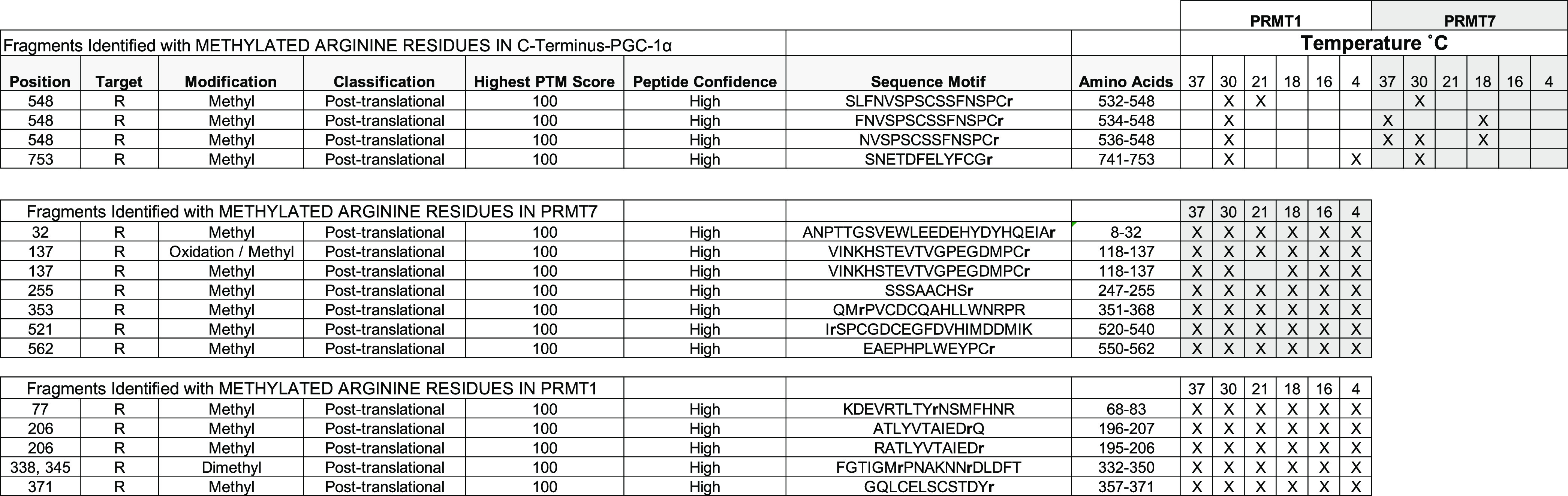
Mass Spectrometry Analyses of Human PGC-1α Methylated at Different Arginine Residues by Two Methyltransferase Enzymes (PRMT1 and PRMT7)^a^

a In vitro methylation reactions were carried out with either recombinant PRMT1 or PRMT7 and PGC-1α (amino acids 481–798) at six different temperatures (37, 30, 21, 18, 16, or 4 °C). Analysis of the controls also revealed automethylation of PRMT7 and PRMT1.

Of the 47 arginine residues located in the C-terminus of the PGC-1α, two methylated arginine residues were identified. Surprisingly, none of the methylated arginine residues fall within the RS regions (565–598 and 617–631). Moreover, only R753 falls within the RRM domain (676–755). Greater methylation at RXR motifs was expected. However, PRMT7 preferentially methylates RXR motifs with adjacent basic residues such as lysine.^18^ The RXR motifs of PGC-1α may not get methylated because they may not contain enough basic residues to accommodate the two acidic amino acids (Asp-147 and Glu-149) located within the PRMT7 enzyme active site necessary for its substrate preference.^17^ Regardless, an initial screen of methylated arginine residues carried out by an ABI-Sciex 5800 MALDI-TOF mass spectrometry revealed methylated sites in PGC-1α. Specifically, a truncated portion of the C-terminus of PGC-1α (aa 573–767) was methylated by PRMT7 (or PRMT1) at 37, 30, 21, 18, and 16 °C. Although the exact arginine residue(s) could not be identified, and this preliminary screen did not return a high confidence score for all values, some of the data suggest that further arginine methylation within amino acid residues 626–677 (containing several RXR motifs) may be possible (Table S2). We reasoned that at its sequence, which contains multiple RXR motifs, there are additional methylated arginine residues yet to be identified in PGC-1α. Thus, we next explored whether PGC-1α would become methylated by other PRMTs using methylation predication algorithms.

**Figure 4:**
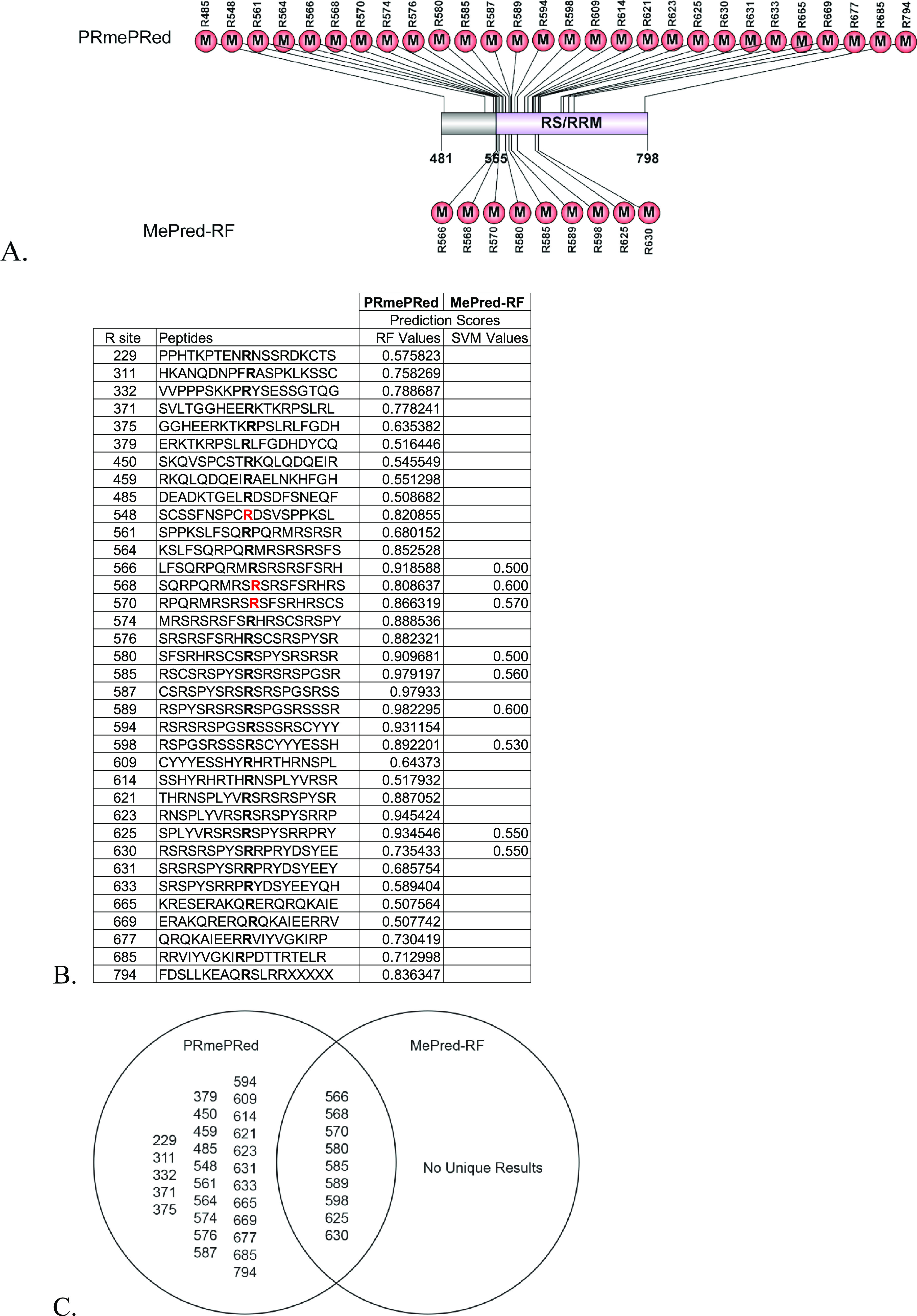
Identification of methylation sites in full-length PGC-1α (UniProt Q9UBK2) using PRmePRed and MePred-RF computational prediction programs. (A) In silico analysis of putative methylation sites in the C-terminus of PGC-1α by PRmePRed (upper methyl groups) and MePred-RF (lower methyl groups); figure created using Illustrator for Biological Sequences (IBS).^41^ (B) RF and SVM scores predicted by the PRmePRed and MePred-RF programs. (C) Venn diagram of the common methylated arginine sites in the middle, predicted by corresponding programs.

Recently, there has been an increased effort to use computational and machine learning techniques based on support vector machines (SVMs) or random forest (RF) algorithms to predict possible methylation sites based on a protein’s sequence and/or structure. These include programs such as PRmePRed^25^ and MePred-RF^26^ which are validated, useful for uncovering putative methylated arginine sites, easy to use, and readily available online. The full-length sequence of PGC-1α was inputted into each of these programs with the expectation that they would confirm methylation of the residues identified experimentally and find novel sites. We note that when only the C-terminus was also inputted into each of these programs, they yielded identical results (data not shown). Figure 4A shows a map of the C-terminus of PGC-1α. The numbers above and below the map show the positions of the predicted methylated arginine sites corresponding to PRmePRed, and MePred-RF methylation prediction programs. PRmePRed uses an SVM-based algorithm, while Me-PredRF uses an RF-based algorithm. Figure 4B lists the RF and SVM prediction scores assigned to each R-site by MePred-RF. A high prediction score indicates a high confidence in the result. The Venn diagram in Figure 4C shows that the SVM algorithm used by PRmePRed predicted the most arginine methylation sites with 36 putative arginine methylation sites, while the RF algorithm used by Me-PredRF was more stringent and predicted only 9 putative arginine methylation sites. Only PRmePRed validated the methylation of R548. None of the algorithms confirmed the methylation of R753. However, we note that these prediction programs do not account for the effects of temperature changes or methylation by different members of the PRMT family. Moreover, both identified novel potential sites.

Accuracy among computational PTM prediction programs is the subject of active investigation and beyond the scope of this work. Despite this, we speculate here about the different results from the two arginine prediction programs. Although both programs use stringent datasets for developing predictor statistics, the initial dataset used for these machine learning algorithms establishes initial parameters and was different for each: MePred-RF employed 2351 total entries (180 positive sequences for arginine methylation), while PRmePRed employed 6837 total entries (1298 positive sequences for arginine methylation). Since the list of experimentally verified methylated arginine residues continues to grow, sequences among the datasets listed as “negative for arginine methylation” may now contain potential sites that could be methylated but that had not been identified or have yet to be identified as methylated arginine sites. Moreover, each of these prediction programs places different values on chemical properties such as charge, hydrophilicity, isoelectric point, and structure within the sequence windows and among the overall protein. For example, whether the arginine residues are solvent-exposed, or whether they are located within a particular secondary structure of the protein aid in making the predictions. Structural, evolutionary, and/or disorder information is not always available. We note here that only PRmePRed validated two (R665 and R669) of the three previously identified arginine methylation sites: R665, R667, and R669.^13^ This may be due to the inherently disordered nature of PGC-1α. Despite this, both programs are useful to screen putative substrates and test biological hypotheses. In addition, each of these programs builds upon the work of Daily et al., who built a predictor for methylation by taking into account intrinsic disorder.^45^ Thus, we pursued our investigation of PGC-1α by considering the postulate that PTMs preferentially occur in intrinsically disordered regions.

Thus far, we identified two methylated arginine residues deposited by two different PRMTs: R548, and R570 by PRMT1 and PRMT7, *in vitro*. We also identified potential arginine residues: R566, R568, R570, R580, R585, R589, R598, R625, and R630, *in silico*, by two independent methylation prediction programs. We next studied the structure of PGC-1α to map the methylated arginine residues so that we may determine possible insights into the function of arginine methylation. To date, a crystal structure of the full-length PGC-1α has not been solved, as determined from Protein Data Bank searches. In addition, PGC-1α has been characterized as an intrinsically disordered protein (IDP).^46^

**Figure 5:**
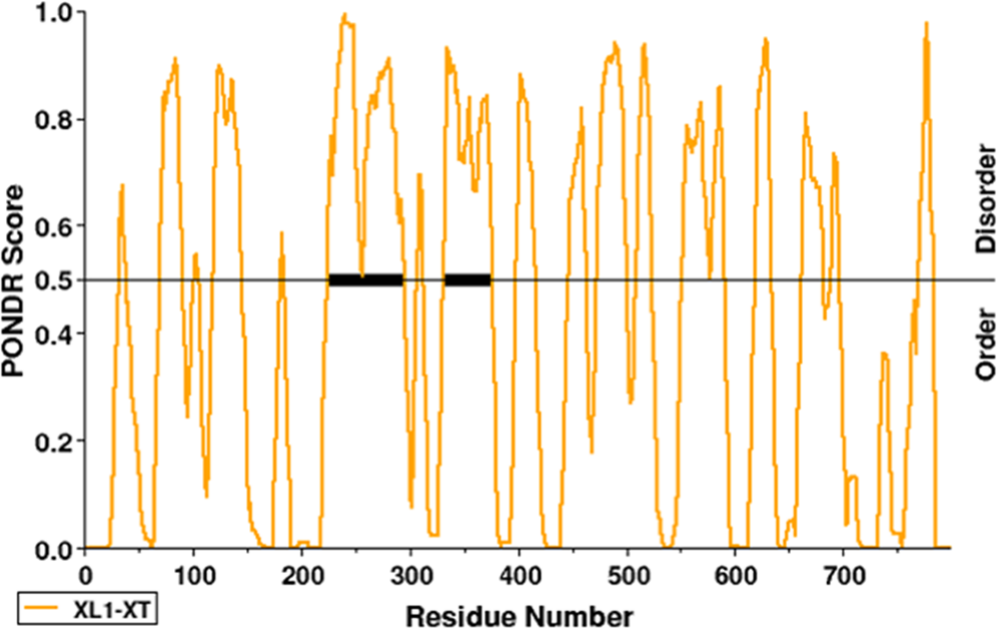
PGC-1α is an intrinsically disordered protein with several methylated arginine residues. PONDR IDP graph showing the disordered and ordered regions of PGC-1α.

IDPs contain flexible regions that facilitate protein–protein interactions and promote access to various PTMs.^47^ In reviewing the many PTMs located on the C-terminus of PGC-1α (Figure 1B) and given its physiological role as a master metabolic regulator, we next analyzed whether arginine methylation sites in PGC-1α are located at intrinsically disordered regions that may affect accessibility. To further understand the function of arginine methylation, we analyzed PGC-1α using PONDR predictor of natural disordered regions.^48^Figure 5 shows a graph of PGC-1α created by PONDR, which determined that PGC-1α contains 18 disordered regions. Specifically, there are 361 disordered residues and a stretch of 71 amino acids (224–294) as the longest disordered region. With respect to the C-terminus, PGC-1α contains the following disordered regions: 473–500, 509–523, 551–576, 578–590, 619–634, 662–681, 689–696. The methylated amino acid residue R570 identified in this study *in vitro*, as well as those previously identified by Teyssier et al., (R665, R667, and R669), fall within these disordered regions. IDPs are also known to interact with binding partners with high specificity, but modest affinity.^49^ Considering its largely unstructured nature, we postulate that PGC-1α is most likely sampling its structure to expose sequences that allow for PTMs such as arginine methylation.

**Figure 6:**
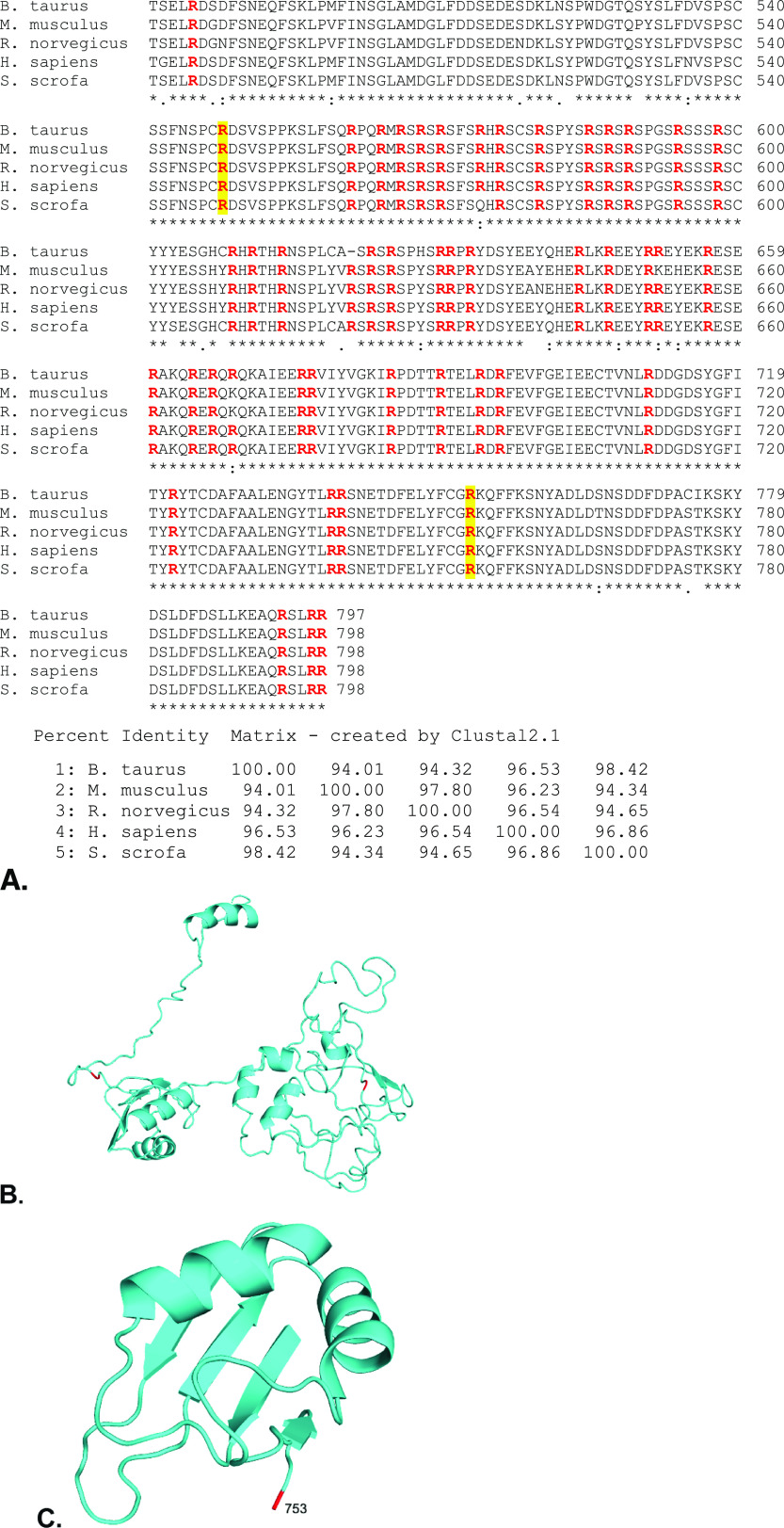
(A) Sequence alignment of the C-terminus of PGC-1α (480–798). Arginine residues confirmed in this study *in vitro*: R548 and R753, shown in red and highlighted in yellow. All other arginine residues are shown in red and boldface. Multiple sequence alignments were generated using Clustal O (1.2.4), percent identity matrix shown below the alignment. (B) Molecular model of the C terminus with methylated arginine groups identified in this study highlighted in red. (C) Magnified portion of the C-terminus containing only the RNA recognition motif (RRM) domain with methylated arginine 753 highlighted in red. Figures for (B) and (C) were generated using Phyre-2 and PyMOL. The Phyre-2 web portal for protein modeling, prediction, and analysis was used to obtain the model and was then imported into PyMOL for structure annotation.^54^

The intrinsically disordered nature of PGC-1α has failed to yield a crystal structure of the full-length protein. Nevertheless, a putative structure can be obtained *via* AlphaFold (https://alphafold.ebi.ac.uk/entry/Q9UBK2).^50,51^ AlphaFold produces a per-residue confidence score (pLDDT) between 0 and 100. The structure for PGC-1α determined by AlphaFold yields very few portions of this protein at high confidence. Moreover, all of the methylated arginine residues identified *in vitro* and *in silico* by this study contain confidence scores below 50, which correlate with disorder. Given the highly conserved nature of PGC-1α in mammals, a sequence alignment is shown in Figure 6A,^52,53^ a putative structure of the C-terminus of PGC-1α was modeled using Phyre-2^54^ and PyMOL (PyMOL Molecular Graphics System, Version 2.0 Schrödinger, LLC), with arginine residues shown in red (Figure 6B).

PGC-1α contains an RNA recognition motif (RRM). RRMs are one of the most common RNA binding domains, responsible for binding to RNA and abundant in intrinsically disordered regions.^55^ We modeled solely the RRM domain of PGC-1α (Figure 6C). Although PGC-1α contains very little structure in the C-terminus, this domain adopts a β_1_α_1_β_2_β_3_α_2_β_4_ topology forming two α-helices against an antiparallel β-sheet consistent with an RRM motif.^56^ The overall flexibility of its C-terminus is consistent with its physiological role as a master regulator and provides a possible explanation for its tissue-specific signaling and responsiveness to temperature changes.

## Conclusions

Tissue-specific, PGC-1α is found wherever energy is needed. Thus, it is expressed in highly oxidative tissues that are rich in mitochondria including embryonic brown adipose tissue, heart, skeletal muscle cells, kidney, and brain.^5,57−61^ According to the tissue where it is expressed, PGC-1α activity is induced by increased energy demand during fasting,^62^ temperature changes,^5,63^ calorie restriction,^64^ and exercise.^65−67^ Several tissue-specific PGC-1α isoforms have been identified, including muscle, liver, and central nervous system (CNS-PGC-1α).^66,68−70^ In addition to transcription, PGC-1α is also regulated by post-translational modifications.

We set out to determine whether PGC-1α is methylated at arginine residues by PRMT7. Both PGC-1α and PRMT7 are highly expressed in skeletal muscle and deletion of *PRMT7* gene causes a decrease in PGC-1α expression.^37^ PRMT7 methylates RXR motifs and has greater activity at or below room temperature. Although we expected to see greater methylation of PGC-1α since it contains many RXR motifs, after performing several *in vitro* methylation reactions and analyses by mass spectrometry, our data show that PGC-1α is methylated at arginine residue 548 and 753 by both PRMT1 and PRMT7 at temperatures at or below 30 °C. Physiological studies that include a temperature dependence may reveal direct interaction between PRMT7 and PGC-1α. We also used computational prediction programs PRmePRed, and MePred-RF to anticipate additional arginine methylation sites. PRmePRed predicted R548 as a site; however, neither program predicted methylation of R753 or R667, a methylated arginine residues previously verified experimentally.^13^ Computational prediction programs rely on experimental results, often MS-based proteomics, as the basis for their algorithms.^25^ While it is possible that some of these predicted arginine sites can be further verified experimentally using other members of the PRMT family, the fact that experimental data is lacking for PGC-1α as an intrinsically disordered protein operating at variable temperatures, may explain why computational predicted residues were not similarly detected experimentally. Additionally, while both of these computational prediction programs used stringent datasets, they may have assigned a disproportionate weight to positive flanking residues that are known to affect substrate binding and catalysis by PRMT active site.^71^ In preparation for this manuscript, we have discovered that a new prediction program that addresses overfitting by early stopping is now available.^72^

The exact role of temperature-dependent arginine methylation of PGC-1α by PRMT7 remains unclear. As an IDP, PGC-1α has many protein partners, which affect its function as a master regulator. In principle, colder temperatures inhibit the rate of chemical reactions. However, the disordered nature of PGC-1α may allow for exposure of specific arginine residues at lower temperatures making them amenable to methylation by PRMT7. This type of plasticity has been observed in other IDPs allowing them to adopt different conformations based on temperature, pH, salinity, *etc*.^73^ In addition, PGC-1α may have specific binding partners with Tudor domains that recognize methylated arginine residues at low temperatures.^74^ Moreover, PGC-1α is methylated at the C-terminus within the RNA recognition motif. It is possible that arginine methylation will disrupt protein–ligand binding with RNA polymerase II, RNA processing factors, or other proteins involved in splicing.^75^ To understand the physiological consequence of these modifications, *in vivo* experiments conducted at varying temperatures are underway.

## Supporting Information Available

List of amino acid sequences of PGC-1α constructs (Table S1); methylation reactions of PGC-1α at various temperatures (Figure S1); and list of peptide sequences analyzed by mass spectrometry (Table S2) (PDF)

## Accession Codes

PRMT1, accession AAH62964, PRMT7, accession NP_663379, AND PGC-1α or PPARGC1A UniProt Q9UBK2
