# Supplementary material for: Arginine Methylation of the PGC-1α C-Terminus Is Temperature-Dependent
Source: Biochemistry. 2022 Dec 19;62(1):22–34. doi: 10.1021/acs.biochem.2c00363 (PMC9813912; doi:10.1021/acs.biochem.2c00363)
Supplement: Supplementary file 1 — bi2c00363_si_001.pdf [file bi2c00363_si_001.pdf]

Arginine methylation of the PGC-1α C-terminus is Temperature Dependent

*Meryl Mendoza[a], Mariel Mendoza[b], Tiffany Lubrino[d], Sidney Briski[d], Immaculeta Osuji[a], Janielle Cuala[a], Brendan Ly[a], Ivan Ocegueda[a], Harvey Peralta[a], Benjamin A. Garcia[c], and Cecilia I. Zurita-Lopez[d]**

*Corresponding author email: ceclopez@chapman.edu

[a]Department of Chemistry and Biochemistry, California State University, Los Angeles, 5151 State University Drive, Los Angeles, CA 90033, [b]Department of Biochemistry and Biophysics, University of Pennsylvania, Philadelphia, PA, 19104, [c]Department of Biochemistry and Molecular Biophysics, Washington University School of Medicine, St. Louis, MO 63110, and [d]Schmid College of Science and Technology, Keck Center for Science and Engineering, Chapman University, 450 N. Center Street, Orange, CA 92866

Table S1. Amino acid sequences of GST-PGC-1α constructs used as substrates for *in vitro* methylation reactions. Arginine residues are in boldface. G1 is the name of a truncated PGC-1α construct that was bacterially expressed and purified (a kind gift from Dr. Michael Stallcup, USC) highlighting mutations (distinguishing it from the WT sequence) (red), (A) a portion of human PGC-1α protein purchased from Creative Biomart (Cat#PPARGC1A-729H, Shirly, NY), and (B) a portion of human PGC-1α protein purchased from Abcam (Cat#ab124541, Cambridge, UK).

Name	Sequence	Mutation	MW
WT	532-<br>SLFNVSPSCSSFNSPCRDSVSPPKSLFSQ**R**PQ**R**M**R**S**R**S**R**SFS**R**H<br>**R**SCS**R**SPYS**R**S**R**S**R**SPGS**R**SSS**R**SCYYYESSHY**R**H**R**TH**R**NSPLY<br>V**R**S**R**S**R**SPYS**RR**P**R**YDSYEE-640	None	
Peptide	551-VSPPKSLFSQ**R**PQ**R**M**R**S**R**S**R**SFS**R**H**R**SCS**R**SPYS**R**S**R**SR**S**<br>-590	None	4.7 kDa
GST-G1	566-<br>**R**S**R**S**R**SFSRHRSCSRSPYSRSRSRSPGSRSSSRSCYYYESSHYR<br>HRTHRNSPLYV**K**S**K**S**K**SPYSRRPRYDYEEY-640	R-K 621, 623, 625	38 kDa
A*	573-<br>SFS**R**H**R**SCS**R**SPYS**R**S**R**S**R**SPGS**R**SSSRSCYYYESSHY**R**H**R**TH**R**<br>NSPLYV**R**S**R**S**R**SPYS**RR**P**R**YDSYEEYQHE**R**LK**R**EEY**RR**EYEK**R**E<br>SE**R**AKQ**R**E**R**Q**R**QKAIEE**RR**VIYVGKI**R**PDTT**R**TEL**R**DRFEVFGE<br>IEECTVNL**R**DDGDSYGFITY**R**YTCDAFAALENGYTL**RR**SNETDF<br>ELYFCG**R**KQFFKSNYADLDSN -767	None	
B	481-<br>TGELRDSDFSNEQFSKLPMFINSGLAMDGLFDDSEDESDKLSYP<br>WDGTQSYSLFNVSPSCSSFNSPC**R**DSVSPPKSLFSQ**R**PQ**R**M**R**S**R**<br>S**R**SFS**R**H**R**SCS**R**SPYS**R**S**R**S**R**SPGS**R**SSS**R**SCYYYESSHY**R**H**R**T<br>H**R**NSPLYV**R**S**R**S**R**SPYS**RR**P**R**YDSYEEYQHE**R**LK**R**EEY**RR**EYEK<br>**R**ESE**R**AKQ**R**E**R**Q**R**QKAIEE**RR**VIYVGKI**R**PDTT**R**TEL**R**D**R**FEVF<br>GEIEECTVNL**R**DDGDSYGFITY**R**YTCDAFAALENGYTL**RR**SNET<br>DFELYFCG**R**KQFFKSNFDPASTKSKYDSLDFDSLLKEAQ**R**SL**RR**<br>-798	None	38 kDa

*According to Creative Biomart accession number for PGC-1α isoform 2 from the NCBI website (NP_037393). This is the same sequence as the one described as UniProt Q9UBK2.

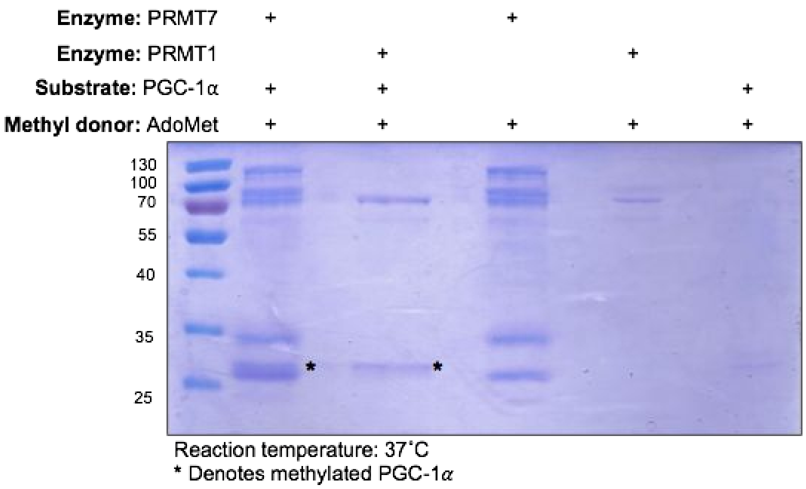
Enzyme: PRMT7
Enzyme: PRMT1
Substrate: PGC-1α
Methyl donor: AdoMet
130
100
70
55
40
35
25
Reaction temperature: 37˚C
* Denotes methylated PGC-1α

A.

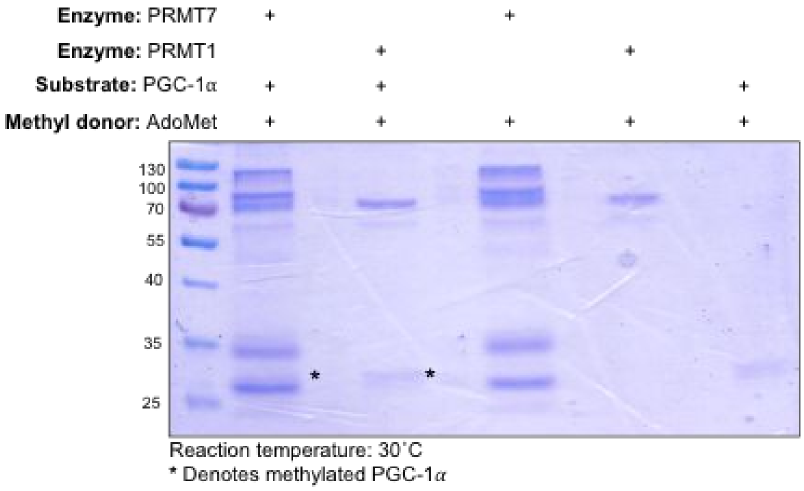
Enzyme: PRMT7
Enzyme: PRMT1
Substrate: PGC-1α
Methyl donor: AdoMet
130
100
70
55
40
35
25
Reaction temperature: 30˚C
* Denotes methylated PGC-1α

B.

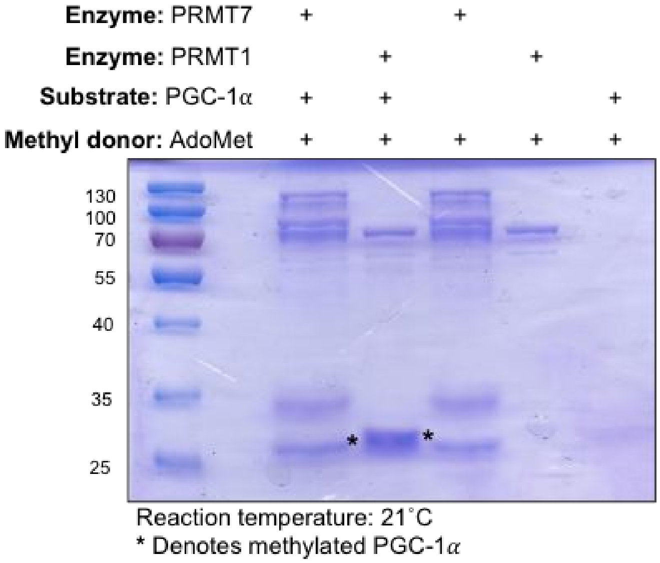
Enzyme: PRMT7
Enzyme: PRMT1
Substrate: PGC-1α
Methyl donor: AdoMet
130
100
70
55
40
35
25
Reaction temperature: 21˚C
* Denotes methylated PGC-1α

C.

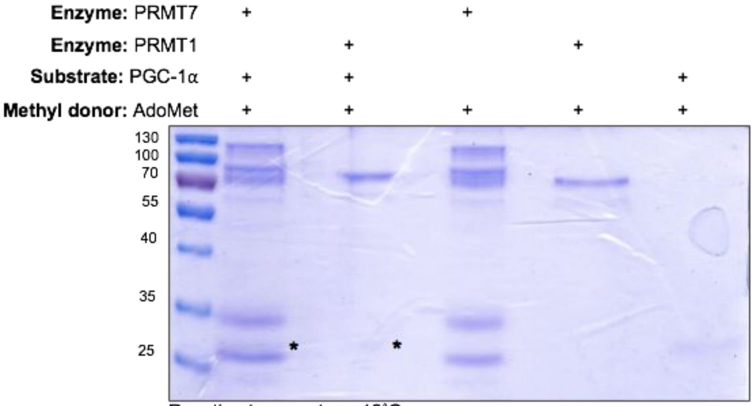

Reaction temperature: 18˚C
* Denotes methylated PGC-1α

D.

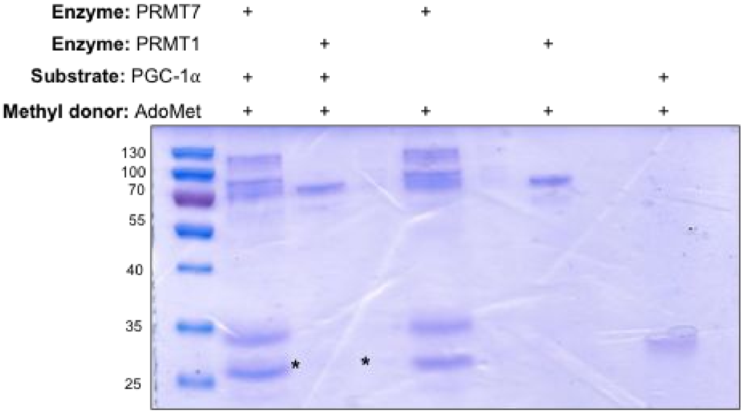

Reaction temperature: 16˚C
* Denotes methylated PGC-1α

E.

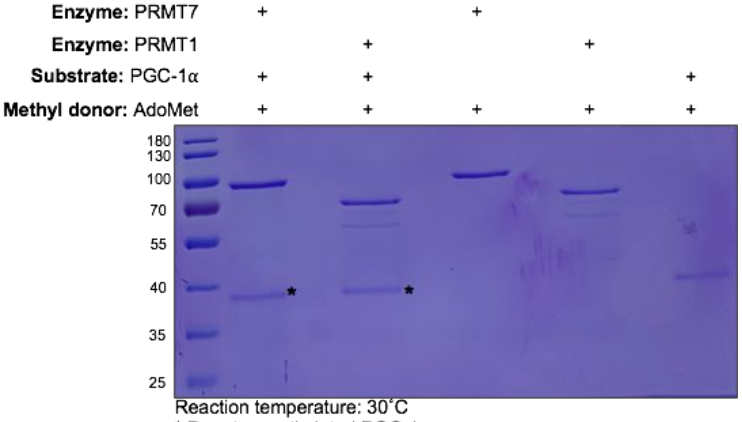

Reaction temperature: 30˚C
* Denotes methylated PGC-1α

F.

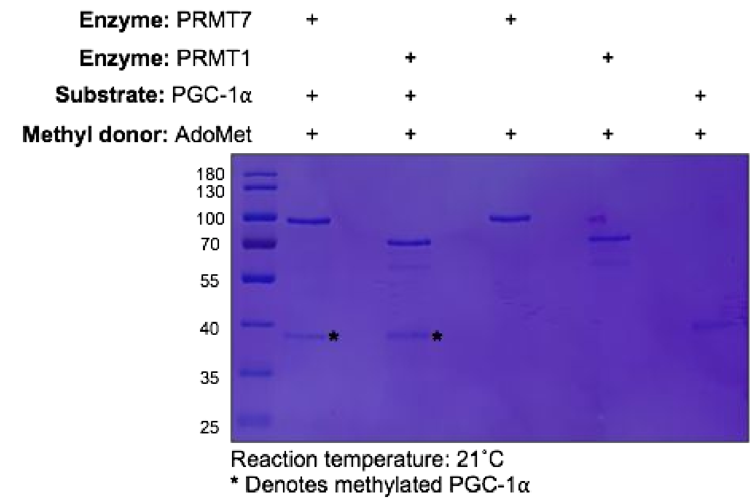
Enzyme: PRMT7 + +
Enzyme: PRMT1 + +
Substrate: PGC-1α + + +
Methyl donor: AdoMet + + + + +
180
130
100
70
55
40
35
25
*
*
Reaction temperature: 21˚C
* Denotes methylated PGC-1α

G.

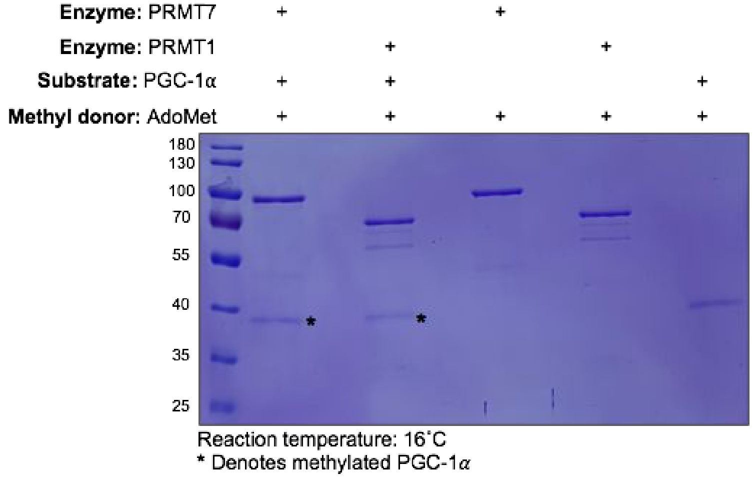
Enzyme: PRMT7 + +
Enzyme: PRMT1 + +
Substrate: PGC-1α + + +
Methyl donor: AdoMet + + + + +
180
130
100
70
55
40
35
25
*
*
Reaction temperature: 16˚C
* Denotes methylated PGC-1α

H.

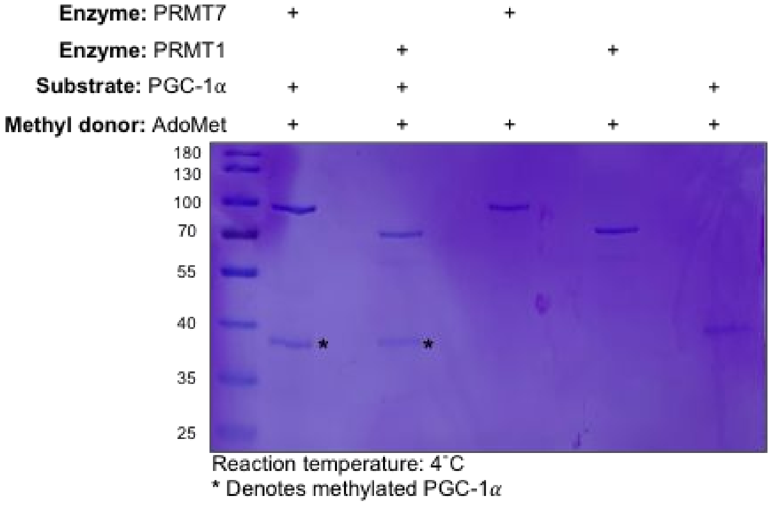
Enzyme: PRMT7 + +
Enzyme: PRMT1 + +
Substrate: PGC-1α + + +
Methyl donor: AdoMet + + + + +
180
130
100
70
55
40
35
25
*
*
Reaction temperature: 4˚C
* Denotes methylated PGC-1α

I.

Figure S1. Methylation reactions using (A-E) PGC-1α (573-767) or (F-I) PGC-1α (481-798) at various temperatures. Both sets of PGC-1α substrates were incubated with preparations of PRMT1 or PRMT7 enzymes (see Materials and Methods). (A-E) PRMT1 or PRMT7 (2 µg) was incubated PGC-1α (2 µg) amino acids 573-767 and 3.2 mM AdoMet with methylation reaction buffer of 500 mM HEPES, 100 mM NaCl, 1 mM DTT, at 37°C, 30°C, 21°C, 18°C, or 16°C for 1h in a final volume of 150 µL master mix these slices were analyzed at UC Irvine Mass spectrometry. (F-K) PGC-1α (3µg) amino acids 481-798 incubated with 1 µg of PRMT1 or PRMT7, 3.2 mM AdoMet, 500 mM HEPES, 100 mM NaCl, 1 mM DTT, at the 37°C, 30 °C, 21 °C, 18 °C, 16 °C, or 4 °C for 1 h in a final volume of 180 µL master mix these slices were analyzed in collaboration with UPENN. All reactions were quenched with 4X loading dye and resolved in a 12% SDS-PAGE gel run at 145 V.

Table S2. List of peptide sequences analyzed by the Mass Spectrometry Facility at UC Irvine using substrate: human recombinant PGC-1α fragment (aa 573-767, Creative Biomart). Either PRMT1 or PRMT7 were incubated with PGC-1α fragment at temperatures 37°C, 30°C, 21°C, 18°C, and 16°C. A) monomethylated (MMA) peptide fragments by PRMT7; B) MMA and asymmetric dimethylated (ADMA) peptide fragments by PRMT1.

A. PRMT7

**37 ˚C**		**30 ˚C**		**21 ˚C**		**18 ˚C**		**16 ˚C**	
**Sample 5**		**Sample 7**		**Sample 9**		**Sample 11**		**Sample 13**	
Peptide Sequence	# of methyl groups	Peptide Sequence	# of methyl groups	Peptide Sequence	# of methyl groups	Peptide Sequence	# of methyl groups	Peptide Sequence	# of methyl groups
626-SPYS**RR**P**R**-633	1							None detected	
626-SPYS**RR**P**R**-633	2								
		622-S**R**S**R**SPYS**RR**P**R**-633	1	622-S**R**S**R**SPYS**RR**P**R**-633	1				
622-S**R**S**R**SPYS**RR**P**R**-633	2			622-S**R**S**R**SPYS**RR**P**R**-633	2	622-S**R**S**R**SPYS**RR**P**R**-633	2		

		652-**R**EYEK**R**ESE**R**-661	2	652-**R**EYEK**R**ESE**R**-661	2				
653-EYEK**R**ESE**R**-661	1					653-EYEK**R**ESE**R**-661	1		

B. PRMT1

**37 ˚C**		**30 ˚C**		**21 ˚C**		**18 ˚C**		**16 ˚C**	
**Sample 6**		**Sample 8**		**Sample 10**		**Sample 12**		**Sample 14**	
Peptide Sequence	# of methyl groups	Peptide Sequence	# of methyl groups	Peptide Sequence	# of methyl groups	Peptide Sequence	# of methyl groups	Peptide Sequence	# of methyl groups
				*610-H**R**TH**R**NSPLYV**R**S**R**-623	1				
None detected		622-S**R**S**R**SPYS**RR**P**R**-633	1	622-S**R**S**R**SPYS**RR**P**R**-633	4				
								626-SPYS**RR**P**R**-633	2
		631-**R**P**R**YDSYEEYQHE**R**-644	1					631-**R**P**R**YDSYEEYQHE**R**-644	1
		631-**R**P**R**YDSYEEYQHE**R**LK-646	3						
				645-LK**R**EEY**RR**EYEK**R**-657	1				
		652-**R**EYEK**R**ESE**R**-661	2	652-**R**EYEK**R**ESE**R**-661	2				

				658-ESE**R**AKQ**R**E**R**Q**R**QK-671	1				
				664-Q**R**E**R**Q**R**-669	2	664-Q**R**E**R**Q**R**-669	2	664-Q**R**E**R**Q**R**-669	2
				666-E**R**Q**R**QKAIEE**RR**-677	3				
